# Supplementary material for: Tumorigenicity decrease in Bcl-xL deficient MDCK cells ensuring the safety for influenza vaccine production
Source: PLoS One. 2024 Dec 16;19(12):e0311069. doi: 10.1371/journal.pone.0311069 (PMC11649150; doi:10.1371/journal.pone.0311069)
Supplement: S1 File — (ZIP) [file pone.0311069.s002.zip › S2_File/4_KOG_enrichment/network/B_vs_A.down_KOG_enrichment_network.pdf]

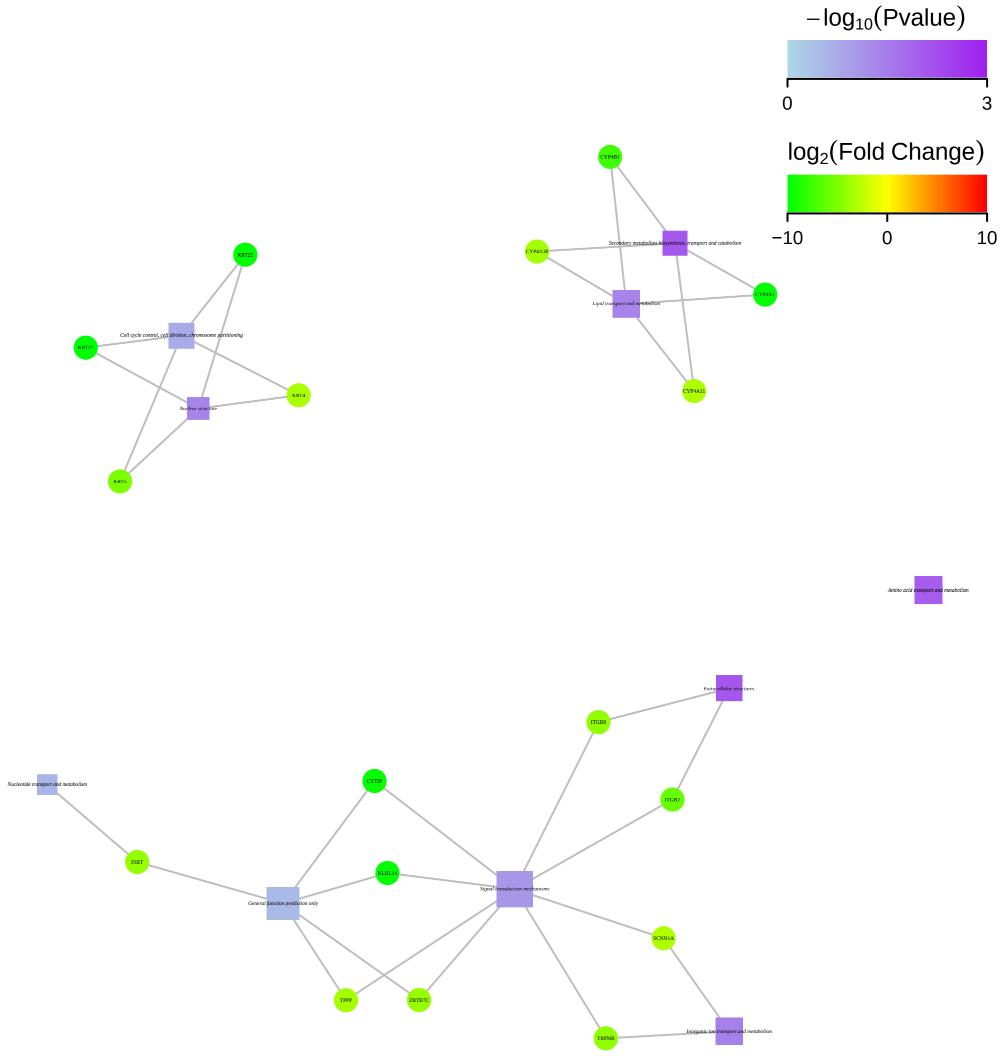

$-\log_{10}(\mathrm{Pvalue})$
0
3
$\log_2(\mathrm{Fold\ Change})$
−10
0
10
KRT25
Cell cycle control, cell division, chromosome partitioning
KRT27
KRT4
Nuclear structure
KRT5
CYP4B1
CYP4A38
Secondary metabolites biosynthesis, transport and catabolism
CYP4X1
Lipid transport and metabolism
CYP4A11
Amino acid transport and metabolism
Extracellular structures
ITGB8
Nucleotide transport and metabolism
CYTIP
ITGB2
FHIT
KLHL14
Signal transduction mechanisms
General function prediction only
SCNN1A
TPPP
ZBTB7C
TRPM8
Inorganic ion transport and metabolism
